# Experiments with Strychnia on the Horse

**Published:** 1850-05

**Authors:** 


*Experiments with Strychnia on the Horse.* By ARCHD. HALL, M. D. Lecturer on Materia Medica, McGill College.

The horse experimented on was 24 years of age, in good health and spirits, the property of Mr. Mason, V. S., who was desirous of killing him, for the purpose of making a skeleton, as well as for pathological as anatomical purposes, as the animal had suffered some years ago from certain diseases implicating some of the joints of both the fore and hind extremities. Mr. Mason offered no objection to, but, on the contrary, did everything to facilitate the experiment. Failing to obtaining *glonoine* for the purposes of the experiment, strychnia was resorted to ; and the object was to determine, if possible, the minimum quantity capable of inducing death. Accordingly, in conjunction with Dr. David, and Messrs. Lyman and Cary, I went to the livery of Mr. Mason, on January 31st, and at 3h. 24m. administered 2 grains of strychnia, dissolved in ℥ ij. of whiskey, and ℥ iv. of water. The pulse, examined before the exhibition of the strychnia, was 40 in the minute, and the animal was in full spirits.

3h 30m. The pulse was now 44. This might have been due to the exercise of walking, which we made him undergo.

3h 35m. No apparent effects perceptible. Two grains more exhibited in the same manner.

3h 41m. Pulse 40.

3h 49m. The horse cheerful, disposed to play, and to kick when teased.

3h 49m. No apparent effects perceptible. Two additional grains were administered under like circumstances to the former ; the horse still as active and fresh as before, the poison not having taken the slightest effect ; kicking and attempting to bite when teased ; and was made to leap several time over a bar placed for the purpose. During the half hour now intervening, he escaped from the yard, and trotted actively up to the Place d'Armes, a distance of about 350 or 400 yards, whither he was followed and brought back. He had now taken six grains of the nia, without the slightest perceptible effect, and I resolved to double the dose now taken. Accordingly at

4h 19m. six additional grains of strychnia were given at one dose, under the same circumstances as the preceding ones, and the eff poison now soon began to manifest themselves.

4h 55m. Slight trembling of the tail. The animal otherwise cheerful.

4h 26m. Trembling of the tail more marked; pulse 60: the animal still disposed to eat.

4h 30m. Belly drawn up; loins hooped; abdominal muscles slightly convulsed, with convulsive twitches of the forelegs, stiffness of the joints, and difficulty of locomotion; the latter apparently depending on mencing muscular rigidity.

4h 33m. Sides and belly as hard as a drum, due to apparent tonic spasm of the abdominal muscles; trembling very much increased, and perceptible over the whole body; tail shaking very much. Pulse 64. Animal still sensible, and disposed to kick when teased, although unable, from the affected condition of the muscles of the hind legs.

4h 36m. The trembling more increased, and the animal evidently in a state of alarm. On attempting to walk, was scarcely able to move a leg. All the other symptoms still existent.

4h 40m. In attempting to reach the stable door, the horse slipped his foot and fell, and shortly afterwards had a general, but not severe, attack of spasm, in which the hind legs seemed chiefly engaged. The respiration was laborious and forced; a copious sweat broke out all over his whole body. His alarm was excessive, almost hydrophopic; the waving of a handkerchief, or even the hand, at the distance of several feet from the eyes, excited an apparent desire of escape; the faculties were therefore still unclouded. Two attempts to raise him to the erect posture were unavailingly made. On the first occasion, he was put on his legs, but immediately fell. In the course of about five minutes, a tetanic spasm affected the whole body, in which, for the first time, the muscles of the face participated. The lips were drawn tightly backwards, the eyeballs retracted; the membrana nictitans closed over the eyes, and gradually returned; the eyeballs became fixed, and at 4h 55m the animal ceased to live, exactly 36 minutes after the exhibition of the six grain dose, and 1h 30m after the exhibition of the first. In all he had taken twelve grains; the first six grains of which did not appear to have been productive of the the slightest apparent influence, even after the lapse of half an hour.

A post mortem examination was out of the question, as the chief parts of interest, viz.; the brain and spinal column, in this examination, would have interfered with Mr. Mason's intentions in the sacrifice of the animal.

Montreal, February, 1850.—*Brit. Amer. Med. Journal.*

